# A Network Analysis of Countries’ Export Flows: Firm Grounds for the Building Blocks of the Economy

**DOI:** 10.1371/journal.pone.0047278

**Published:** 2012-10-19

**Authors:** Guido Caldarelli, Matthieu Cristelli, Andrea Gabrielli, Luciano Pietronero, Antonio Scala, Andrea Tacchella

**Affiliations:** 1 IMT - Institutions Market Technology, Lucca, Italy; 2 ISC-CNR - Institute of Complex Systems, Rome, Italy; 3 LIMS - London Institute for Mathematical Sciences, London, United Kingdom; 4 Department of Physics, University of Rome “Sapienza”, Rome, Italy; Indiana University, United States of America

## Abstract

In this paper we analyze the bipartite network of countries and products from UN data on country production. We define the country-country and product-product projected networks and introduce a novel method of filtering information based on elements’ similarity. As a result we find that country clustering reveals unexpected socio-geographic links among the most competing countries. On the same footings the products clustering can be efficiently used for a bottom-up classification of produced goods. Furthermore we mathematically reformulate the “reflections method” introduced by Hidalgo and Hausmann as a fixpoint problem; such formulation highlights some conceptual weaknesses of the approach. To overcome such an issue, we introduce an alternative methodology (based on biased Markov chains) that allows to rank countries in a conceptually consistent way. Our analysis uncovers a strong non-linear interaction between the diversification of a country and the ubiquity of its products, thus suggesting the possible need of moving towards more efficient and direct non-linear fixpoint algorithms to rank countries and products in the global market.

## Introduction

### Complex Networks

Networks emerged in the recent years as the main mathematical tool for the description of complex systems. In particular, the mathematical framework of graph theory made possible to extract relevant information from different biological and social systems [1]–[3]. In this paper we use some concepts of network theory to address the problem of economic complexity [4]–[7].

Our activity is in the track of a long-standing interaction between economics and physical sciences [8]–[12] and it explains, extends and complements a recent analysis done on the network of trades between nations [13], [14]. Hidalgo and Hausmann (HH) address the problem of competitiveness and robustness of different countries in the global economy by studying the differences in the Gross Domestic Product and assuming that the development of a country is related to different “capabilities”. While countries cannot directly trade capabilities, it is the specific combination of those capabilities that results in different products traded. More capabilities are supposed to bring higher returns and the accumulation of new capabilities provides an exponentially growing advantage. Therefore the origin of the differences in the wealth of countries can be inferred by the record of trading activities analyzed as the expressions of the capabilities of the countries.

### Revealed Competitive Advantage and the country-product Matrix

We consider here the Standard Trade Classification data for the years in the interval 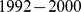. In the following we shall analyze the year 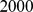, but similar results apply for the other snapshots. For the year 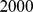 the data provides information on 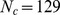 different countries and 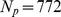 different products.

To make a fair comparison between the trades, it is useful to employ Balassa’s Revealed Comparative Advantage (RCA) [15] i.e. the ratio between the export share of product 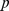 in country 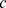 and the share of product 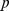 in the world market
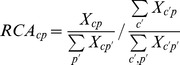
(1)where 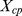 represents the dollar exports of country 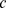 in product 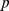.

We consider country 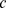 to be a competitive exporter of product 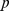 if its RCA is larger than some threshold value, which we take as 1 as in standard economics literature; previous studies have verified that small variations around such threshold do not qualitatively change the results.

The network structure of the country-product competition is given by the semipositive matrix 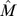 defined as
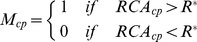
(2)where 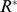 is the threshold (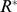 = 1).

To such matrix 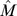 we can associate a graph whose nodes are divided into two sets 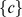 of 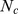 nodes (the countries) and 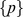 of 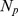 nodes (the products) where a link between a node 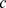 and a node 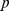 exists if and only if 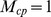, i.e. a bipartite graph. The matrix 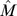 is strictly related to the adjacency matrix of the country-product bipartite network.

The fundamental structure of the matrix 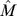 is revealed by ordering the rows of the matrix by the number of exported products and the columns by the number of exporting countries: doing so, 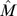 assumes a substantially triangular structure. Such structure reflects the fact that some countries export a large fraction of all products (highly diversified countries), and some products appear to be exported by most countries (ubiquitous products). Moreover, the countries that export few products tend to export only ubiquitous products, while highly diversified countries are the only ones to export the products that only few other countries export.

This triangular structure is therefore revealing us that there is a systematic relationship between the diversification of countries and the ubiquity of the products they make. Poorly diversified countries have a revealed comparative advantage (RCA) almost exclusively in ubiquitous products, whereas the most diversified countries appear to be the only ones with RCAs in the less ubiquitous products which in general are of higher value on the market. It is therefore plausible that such structure reflects a ranking among the nations.

The fact that the matrix is triangular rather than block-diagonal suggests that some successful countries are more diversified than expected. Countries add more new products to the export mix while keeping, at the same time, their traditional productions. The structure of 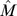 therefore contradicts most of classical macro-economical models that always predict a specialization of countries in particular sectors of production (i.e. countries should aggregate in communities producing similar goods) that would result in a more or less block-diagonal matrix 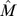.

In the following, we are going to analyze the economical consequences of the structure of the bipartite country-product graph described by 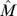. In particular, we analyze the community structure induced by 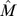 on the countries and products projected networks. As a second step, we reformulate as a linear fixpoint algorithm the HH’s *reflection method* to determine the countries and products respective rankings induced by 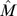. In this way we are able to clarify the critical aspects of this method and its mathematical weakness. Finally, to assign proper weights to the countries, we formulate a mathematically well defined biased Markov chain process on the country-product network; to account for the bipartite structure of the network, we introduce a two parameter bias in this method. To select the optimal bias, we compare the results of our algorithm with a standard economic indicator, the gross domestic product 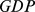. The optimal values of the parameters suggests a highly non-linear interaction between the number of different products produced by each country (*diversification*) and the number of different countries producing each product (*ubiquity*) in determining the competitiveness of countries and products. This fact suggests that, to better capture the essential features of economical competition of countries, we need a more direct and efficient non-linear approach.

## Results

### The Network of Countries

**Figure 1:**
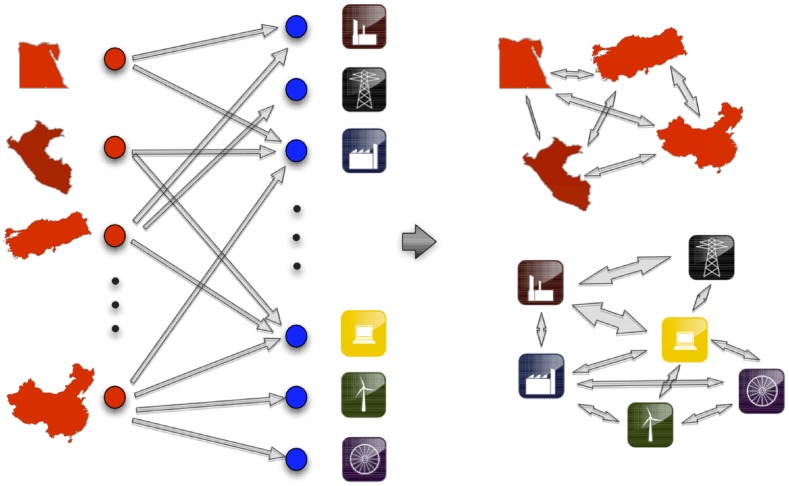
The network of countries and products and the two possible projections.

In order to obtain an immediate understanding of the economic relations between countries induced by their products a possible approach is to define a projection graph obtained from the original set of bipartite relations represented by the matrix 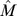 [16]. The idea is to connect the various countries with a link whose strength is given by the number of products they mutually produce. In such a way the information stored in the matrix 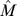 is projected into the network of countries as shown in Fig. 1.

The country network can be characterized by the 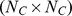 *country-country* matrix 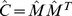. The non-diagonal elements 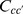 correspond to the number of products that countries 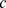 and 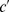 have in common (i.e. are produced by both countries). They are a measure of their mutual competition, allowing a quantitative comparison between economic and financial systems [17]; the diagonal elements 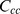 corresponds to the number of products produced by country 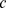 and are a measure of the diversification of country 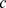.

To quantify the competition among two countries, we can define the similarity matrix among countries as
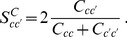
(3)


Note that 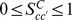 and that small (large) values indicate small (large) correlations between the products of the two countries 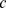 and 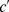. Similar approaches to define a correlation between vertices or a distance [18] have often been employed in the field of complex networks, for example to detect protein correlations [19] or to characterize the interdependencies among clinical traits of the orofacial system [20], [21].

The first problem for large correlation networks is how to visualize the relevant structure. The simplest approach to visualize the most similar vertices is realized by building a Minimal Spanning Tree (MST) [22], [23]. In this method, starting from an empty graph, edges 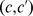 are added in order of decreasing similarity until all the nodes are connected; to obtain a tree, edges that would introduce a loop are discarded. A further problem is to split the graph in smaller sub-graphs (communities) that share important common feature, i.e. have strong correlations. *Similarity*, like analogous correlation indicators, can be used to detect the inner structure of a network; while different methods for community detection vary in their detailed implementation [24], [25], they give reasonably similar qualitative results when the indicators contain the same information.

The MST method can be thus generalized in order to detect the presence of communities by adding the extra condition that no edge between two nodes that have been already connected to some other node is allowed. In this way we obtain a set of disconnected sub-trees (i.e. a forest) embedded in the MST. This *Minimal Spanning Forest* (MSF) method naturally splits the network of countries into separate subsets. This method allows for the visualization of correlations in a large network and at the same time performs a sort of community detection if not precise, certainly very fast.

**Figure 2:**
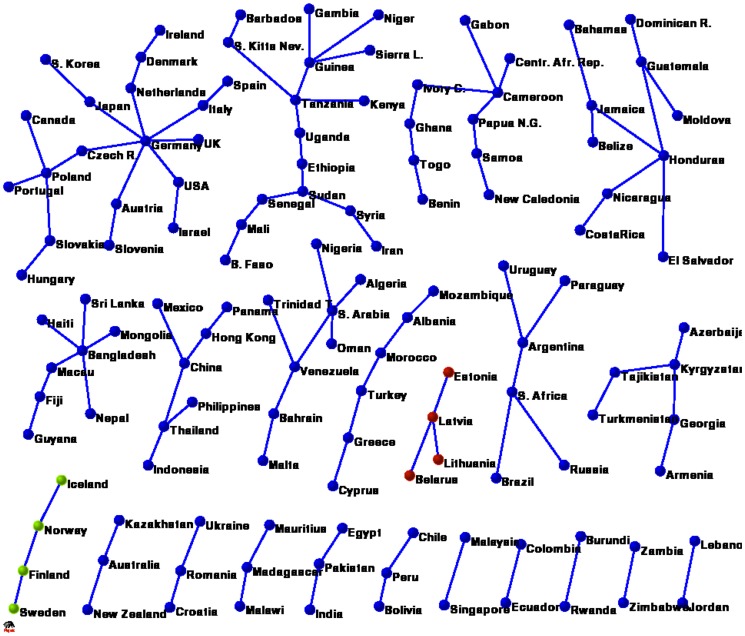
The Minimal Spanning Forest for the Countries. The various subgraphs have a distinct geographical similarity. We show in green northern European countries and in red the “Baltic” republics. In general neighboring (also in a social and cultural sense) countries compete for the production of similar goods.

By visual inspection in Fig. 2 we can spot a large subtree composed by developed countries and some other subtrees in which clear geographical correlations are present. Notice that each subtree contains countries with very similar products, i.e. countries that are competing on the same markets. In particular, developing countries seem to be mostly direct competitors of their geographical neighbors. This features despite its high frequency in most geographical areas, comes unexpected since it is not the most rationale choice [26], [27]: as an example, both banks [28] and countries [29] trade preferentially with similar partners, thereby affecting the whole robustness of the system [30], [31]. This behavior can be reproduced by simple statistical models based on agents’ fitnesses [32], [33].

### The Network of Products

Similarly to countries, we can project the bipartite graph into a product network by connecting two products if they are produced by the same one or more countries and giving a weight to this link proportional to the number of countries producing both products. Such network can be represented by the 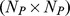 *product-product* matrix 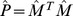. The non-diagonal elements 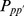 correspond to the number of countries producing both 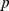 and 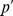 have in common, while the diagonal elements 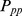 corresponds to the number of countries producing 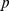.

In analogy with Eq. (3), the similarity matrix among products is defined as
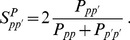
(4)


It indicates how much products are correlated on a market: a value 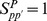 indicates that whenever product 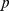 is present on the market of a country, also product 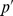 would be present. This could be for example the case of two products 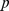, 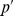 that are both necessary for the same and only industrial process.

**Figure 3:**
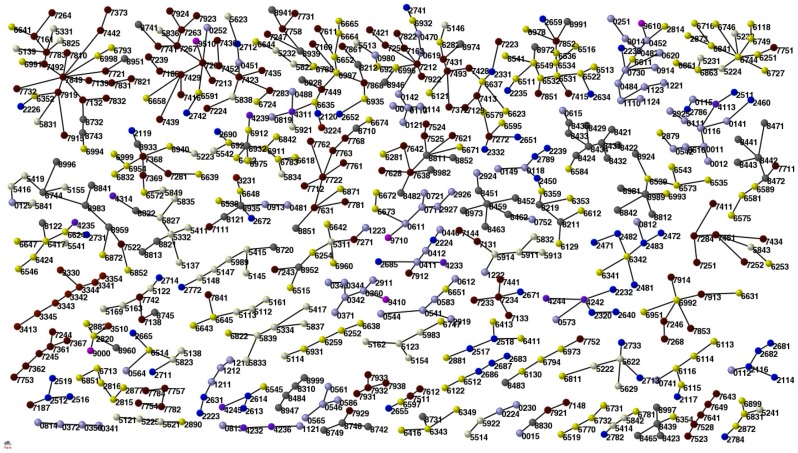
The Minimal Spanning Forest (MSF) for the Products. We put a different color according to the first digit used in COMTRADE classification. This analysis should reveal correlation between different but similar products.

**Figure 4:**
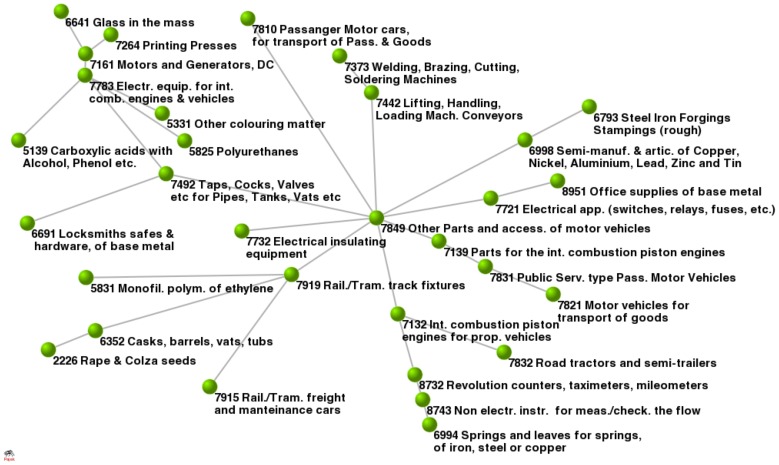
The largest tree in the Products MSF. When passing from classification colors to the real products name, we see they are all strongly related. It is interesting the presence of colza seeds in the lower left corner of the figure.

As in the case of countries, the MSF algorithm can be applied to visualize correlations and detect communities. In the case of the product network this analysis brings to an apparently contradictory results: let’s see why. Products are officially characterized by a hierarchical topology assigned by UN. Within this classification similar issue as “metalliferous ores and metal scraps” (groups 27.xx) are in a totally different section with respect to “non ferrous metals” (groups 68.xx). By applying our new algorithm, based on the economical competition network 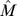, one would naively expect that products belonging to the same UN hierarchy should belong to the same community and *vice-versa*; therefore, if we would assign different colors to different UN hierarchies, one would expect all the nodes belonging to a single community to be of the same color. In Fig. 3 we show that this is not the case. Such a paradox can be understood by analyzing in closer detail the detected communities with the MSF method. As an example, we show in Fig. 4 a large community where most of the vertices belong to the area of “vehicle part and constituents”. In this cluster we can spot the noticeable presence of a vertex belonging to “food” hierarchy. This apparent contradiction is solved up by noticing that such vertex refers to colza seeds, a typical plant recently used mostly for bio-fuels and not for alimentation: our MSF method has correctly positioned this “food” product in the “vehicle” cluster. Therefore, methods based on community detection could be considered as a possible rational substitute for current top-down “human-made” taxonomies [32].

### Ranking Countries and Products by Reflection Method

Hidalgo and Haussman (HH) have introduced in [13], [14] the fundamental idea that the complex set of capabilities of countries (in general hardly comparable between different countries) can be inferred from the structure of matrix 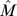 (that we can observe). In this spirit, ubiquitous products require few capabilities and can be produced by most countries, while diversified countries possess many capabilities allowing to produce most products. Therefore, the most diversified countries are expected to be amongst the top ones in the global competition; on the same footing ubiquitous products are likely to correspond to low-quality products.

In order to refine such intuitions in a quantitative ranking among countries and products, the authors of [13], [14] have introduced two quantities: the 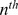 level *diversification* 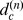 (called 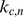 in [13], [14]) of the country 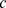 and the 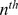 level *ubiquity* 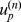 (called 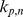 in [13], [14]) of the product 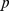. At the zero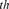 order the diversification of a country is simply defined as the number of its products or
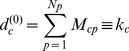
(5)where 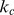 is the degree of the node 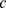 in the bipartite country-product network); analogously the zero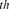 order ubiquity of a product is defined as the number of different countries producing it
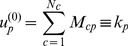
(6)where 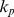 is the degree of the node 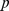 in the bipartite country-product network. The diversification 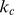 is intended to represent the zero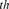 order measure of the “quality” of the country 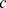 with the idea that the more products a country exports the strongest its position on the marker. The ubiquity 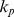 is intended to represent the zero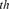 order measure of the “dis-value of the product 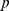 in the global competition with the idea that the more countries produce a product, the least is its value on the market.

In the original approach these two initial quantities are refined in an iterative way via the so-called “reflections method”, consisting in defining the diversification of a country at the 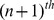 iteration as the average ubiquity of its product at the 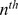 iteration and the ubiquity of a country at the 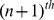 iteration as the average diversification of its producing countries at the 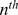 iteration:
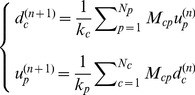
(7)


In vectorial form, this can be cast in the following form
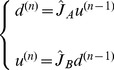
(8)where 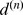 is the 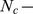
dimensional vector of components 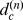, 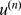 is the 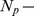
dimensional vector of components 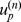, and where we have called 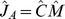 and 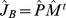 (the upper suffix 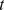 stands for “transpose”), with 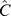 and 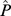 respectively the 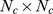 and 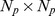 square diagonal matrices defined by 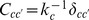 and 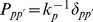.

Such an approach suffers from some problems. The first one is related to the fact that the process is defined in a bipartite networks and therefore even and odd iterations have different meanings. In fact, let us consider the diversification 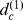 of the 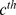 country: as prescribed by the algorithm, 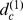 is the average ubiquity of the products of the 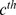 country at the 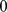-th iteration. Therefore countries with most ubiquitous (less valuable) products would get an highest 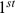 order diversification. On the other hand, the approximately triangular structure of 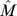 tells us that these countries are the same ones with a small degree and therefore with a low value of the 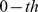 order diversification 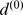. As shown to by [13], [14], this is the case also to higher orders; therefore the diversifications at even and odd iterations are substantially an anti-correlated. Conversely, successive even iterations are positively correlated so that 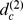 looks a refinement of 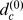, 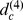 a refinement of 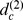 and so on. Same considerations apply to the iterations for the ubiquity of products.

The major problem in the HH algorithm is that it is a case of a consensus dynamics [34], i.e. the state of a node at iteration 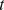 is just the average of the state of its neighbors at iteration 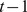. It is well known that such iterations have the uniform state (all the nodes equal) as the natural fixpoint. It is therefore puzzling how such “equalizing” procedure could lead to any form of ranking. To solve such a puzzle, let’s write the HH algorithm as a simple iterative linear system and analyze its behavior.

Focusing only on even iterations and on diversifications, we can write HH procedure as:
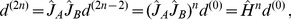
(9)where 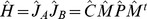 is a 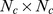 squared matrix.

The matrix 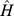 in Eq.9 is a Markovian stochastic matrix when it acts *from the right* on positive vectors, in the sense that every element 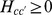 and
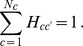



In particular for the given 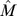 adjacency matrix it is also ergodic. Therefore, its spectrum of eigenvalues is bounded in absolute value by its unique upper eigenvalue 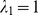. Since 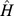 acts on 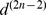 from the left, the right eigenvector 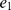 corresponding to the largest eigenvalue 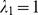 is simply a uniform vector with identical components, i.e. in the 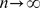 limit 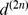 converges to the fixpoint 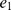 where all countries have the same asymptotic diversification.

It is therefore not a case that HH prescribe to stop their algorithm at a finite number of iterations and that they introduce as a recipe to consider as the ranking of a country the rescaled version of the 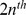 level diversifications [14]

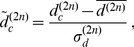
(10)where 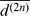 is the arithmetic mean of all 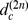 and 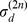 the standard deviation of the same set. With these prescription, HH algorithm seems to converge to an approximately constant value after 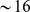 steps.

This observed behavior can be easily be explained by noticing that, in contrast with the erroneous statement in [14], finding the fitness by the reflection method can be reformulated as a fix-point problem (our Eq. 9) and solved using the spectral properties of a linear system. In fact,since the ergodic Markovian nature of 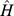 we can order eigenvalues/eigenvectors such that 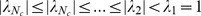. Therefore, expanding 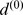 in terms of the right eigenvectors 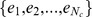 of 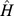 the initial condition
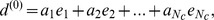
we can write the 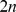-th iterate as



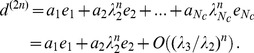
(11)Therefore, at sufficiently large 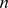 the ordering of the countries is completely determined by the components of 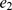; notice that such an asymptotic ordering is independent from the initial condition 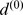 and therefore should be considered as the appropriate fixpoint renormalized fitness 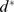 for all countries.

What happens to the HH scheme? At sufficiently large 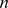, 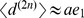 and 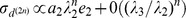; therefore 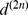 becomes proportional to 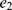 (Eq. 10). The number of iterations 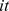 needed to converge is given by the ratio between 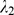 and 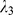 (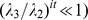; therefore the 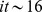 iterations prescribed by HH are not a general prescription but depend on the spectrum of the network analyzed.

Notice also that when the numerical reflection method is used, the renormalized fitness represents a deviation 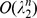 from a constant and can be detected only if it is bigger than the numerical error; therefore only “not too big” 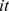 can be employed. On the other hand, the spectral characterization we propose does not suffer from such a pitfall even when. Similar considerations can be developed for the even iterations of the reflection method for the products.

### Biased Markov Chain Approach and Non-linear Interactions

Having assessed the problems of HH’s method, we investigate the possibility of defining alternative linear algorithms able to implement similar economical intuitions about the ranking of the countries while keeping a more robust mathematical foundation. In formulating such a new scheme we will keep the approximation of linearity for the iterations even though we shall find in the results hints of the non-linear nature of the problem.

Our approach is inspired to the well-known PageRank algorithm [35]. PageRank (named after the WWW, where vertices are the pages) is one of the most famous of Bonacich centrality measures [36]. In the original PageRank method the ranking of a vertex is proportional to the time spent on it by an unbiased random walker (in different contexts [11] analogous measures assess the stability of a firm in a business firm network).

We define the weights of vertices to be proportional to the time that an *appropriately biased* random walker on the network spends on them in the large time limit [37]. As shown below, such weights, being the generalization of 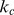 and 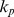, give a measure respectively of competitiveness of countries and “dis-quality” (or lack of competitiveness) of products. As the nodes of our bipartite network are entities that are logically and conceptually separated (countries and products), we assign to the random walker a different bias when jumping from countries to products respect to jumping from products to countries.

Let us call 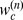 weight of country 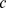 at the 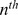 iteration and 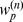 fitness of product 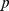 at the 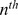 iteration. We define the following Markov process on the country-product bipartite network
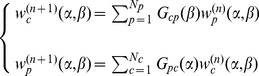
(12)where the Markov transition matrix 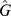 is given by



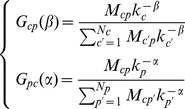
(13)Here 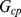 gives the probability to jump from product 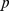 to country 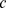 in a single step, and 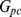 the probability to jump from country 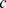 to product 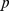 also in a single step. Note that Eqs.(13) define a 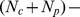
dimensional connected Markov chain of period two. Therefore, random walkers initially starting from countries, will be found on products at odd steps and on countries at even ones; the reverse happens for random walkers starting from products. By considering separately the random walkers starting from countries and from products, we can reduce this Markov chain to two ergodic Markov chains of respective dimension 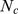 and 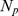. In particular, if the walker starts from a country, using a vectorial formalism, we can write for the weights of countries
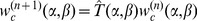
(14)where the 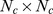 ergodic stochastic matrix 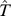 is defined by



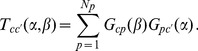
(15)At the same time for products we can write
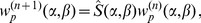
(16)where the 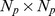 ergodic stochastic matrix 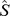 is given by



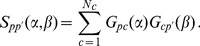
(17)Given the structure of 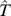 and 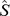, it is simple to show that the two matrices share the same spectrum which is upper bounded in modulus by the unique eigenvalue 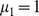. For both matrices, the eigenvectors corresponding to 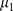 are the stationary and asymptotic weights 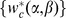 and 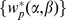 of the Markov chains. In order to find analytically such asymptotic values, we apply the detailed balance condition:
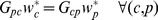
(18)which gives
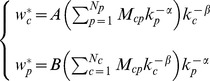
(19)where 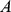 and 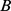 are normalization constants. Note that for 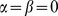 Eq. (13) gives the completely unbiased random walk for which 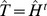 where 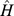 is given in Eq. (9). Therefore, in this case Eqs. (19) become
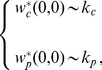
(20)as for the case of unbiased random walks on a simple connected network the asymptotic weight of a node is proportional to its connectivity. Thus, in the case of 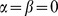 we recover the zero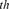 order iteration of the HH’s reflection method. Note that, in the same spirit of HH, 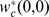 gives a rough measure of the competitiveness of country 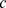 while 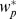 gives an approximate measure of the dis-quality in the market of product 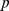. By continuity, we associate the same meaning of competitiveness/disquality to the stationary states 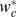/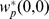 at different values of 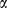 and 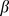.

**Figure 5:**
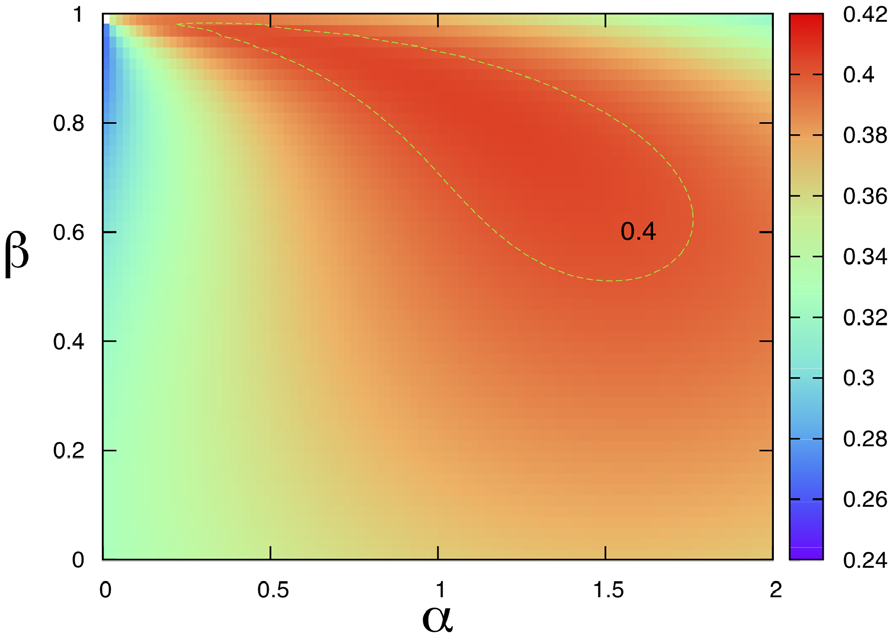
The plot of the mean Correlation (square of Pearson coefficient, 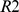) between logarithm of GDP and fixpoint weights of countries in the biased (Markovian) random walk method as a function of parameters 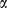 and 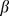. The contour plot for a level of 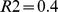 is indicated as a green loop in the orange region (year 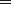 1998).

To understand the behavior of our ranking respect to the bias, we have analyzed the mean correlation (square of the Pearson coefficient) for the year 1998 (other years give analogous results) between the logarithm of the GDP of each country and its weight (Eqs. (19) for different values of 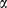 and 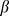 (see Fig. 5). We are aware that GDP is not an absolute measure of wealth [38] as it does not account directly for relevant quantities like the wealth due to natural resources [39]. Nevertheless, we expect GDP to monotonically increase with the wealth. What network analysis shows is that the number of products is correlated with both quantities. We envisage such kind of analysis in order to define suitable policies for underdeveloped countries [40].

It is interesting to note that the region of large correlations (region inside the contour plot in the Fig. 5) is found in the positive quadrant for about 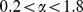 and 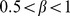; in particular the maximal value is approximately at 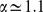 and 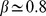. These results can be connected with the approximately “triangular” shape of the matrix 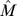. In fact, let us rewrite Eqs. (19) (apart from the normalization constant) as:
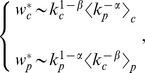
where 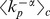 is the arithmetic average of 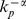 of the products exported by country 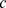 and 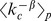 is the arithmetic average of 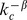 for countries exporting product 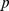. Since 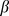 is substantially positive and slightly smaller of 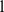 and 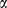 is definitely positive with optimal values around 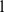, the competitive countries will be characterized by a good balance between a high value of 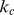 and a small typical value of 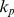 of its products. Nevertheless, since the optimal values of 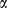 are distributed up to the region of values much larger than 1 (i.e. 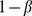 is significantly smaller than 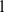), we see that the major role for the asymptotic weight of a country is played by the presence in its portfolio of un-ubiquitous products which alone give the dominant contribution to 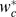. A similar reasoning leads to the conclusion that the dis-value of a product is basically determined by the presence in the set of its producers of poorly diversified countries that are basically exporting only products characterized by a low level of complexity.

Our new approach based on biased Markov chain theory permits thus to implement the interesting ideas developed by HH in [14] on a more solid mathematical basis using the framework of linear iterated transformations and avoiding the indicated flaws of HH’s “reflection method”. Interestingly, our results reveal a strongly non-linear entanglement between the two basic information one can extract from the matrix 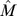: diversification of countries and ubiquity of products. In particular, this non-linear relation makes explicit an almost extremal influence of ubiquity of products on the competitiveness of a country in the global market: having “good” or complex products in the portfolio is more important than to have many products of poor value. Furthermore, the information that a product has among its producers some poorly diversified countries is nearly sufficient to say that it is a non-complex (dis-valuable) product in the market. This strongly non-linear entanglement between diversifications of countries and ubiquities of products is an indication of the necessity to go beyond the linear approach in order to introduce more sound and direct description of the competition of countries and products possibly based on a suitable *ab initio* non-linear approach characterized by a smaller number of *ad hoc* assumptions [41].

**Figure 6:**
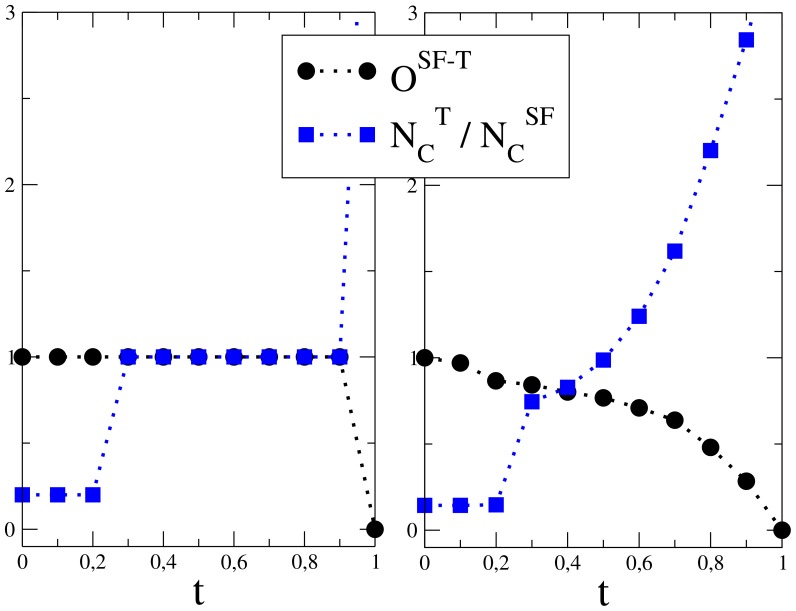
Graphs of the overlap 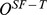 between the spanning forest and threshold graph and the ratio 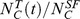 versus the threshold 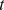. Here 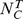 is the number of clusters in the threshold graph and 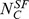 is the number of clusters in the spanning forest. (left panel:) Curves for 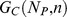. In this deterministic case, 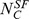 equals the number of communities and both curves intersect when the 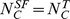. (right panel:) Curves for 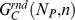; curves are averaged over 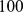 configurations of the noise.

## Discussion

In this paper we applied methods of graph theory to the analysis of the economic productions of countries. The information is available in the form of an 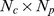 rectangular matrix 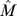 giving the different production of the possible 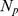 goods for each of the 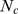 countries. The matrix 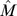 corresponds to a bipartite graph, the country-product network, that can be projected into the country-country network 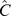 and the product-product network 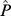. By using complex-networks analysis, we can attain an effective filtering of the information contained in 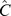 and 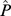. We introduce a new filtering algorithm that identifies communities of countries with similar production. As an unexpected result, this analysis shows that neighboring countries tend to compete over the same markets instead of diversifying. We also show that a classification of goods based on such filtering provides an alternative product taxonomy determined by the countries’ activity. We then study the ranking of the countries induced by the country-product bipartite network. We first show that HH’s ranking is the fix-point of a linear process; in this way we can avoid some logical and numerical pitfalls and clarify some of its weak theoretical points. Finally, in analogy with the Google PageRank algorithm, we define a biased, two parameters Markov chain algorithm to assign ranking weights to countries and products by taking into account the structure of the adjacency matrix of the country-product bipartite network. By correlating the fix-point ranking (i.e. competitiveness of countries and products) with the GDP of each country, we find that the optimal bias parameters of the algorithm indicate a strongly non-linear interaction between the diversification of the countries and the ubiquity of the products. The fact that we still find some discrepancies between fitnesses and GDP is related to the fact that they measure related but different things. In particular while GDP is a measure of the richness of a country, the fitness measures the possibility of a certain country to sustain its growth or to recover from crises.

**Table 1:** Example of estimates of the number of communities for the noisy case; notice that 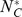 is close to the expected value 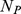.

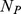	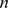	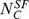	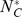
10	5	14	11.1
9	5	13	10.1
7	5	9	7.2
5	10	12	6.2
5	7	8	6.5
5	5	7	5.3

Intersection point between 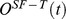 and 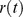 are calculated averaging curves over 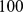 random samples.

## Materials and Methods

### Graphs

A graph is a couple 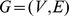 where 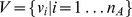 is the set of vertices, and 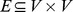 is the set of edges. A graph 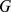 can be represented via its adjacency matrix 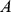.
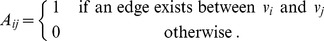
(21)


The degree 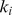 of the node 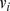 is the number 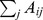 of its neighbors.

An unbiased random walk on a graph 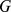 is characterized by a probability 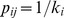 of jumping from a vertex 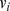 to one of its 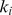 neighbors and is described by the jump matrix
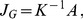
(22)where 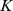 is the diagonal matrix 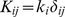 corresponding to the nodes degrees.

### Bipartite Graphs

A bipartite graph is a triple 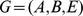 where 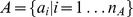 and 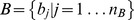 are two disjoint sets of vertices, and 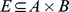 is the set of edges, i.e. edges exist only between vertices of the two different sets 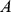 and 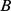.

The bipartite graph 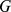 can be described by the matrix 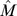 defined as
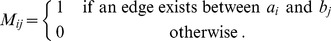
(23)


In terms of 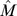, it is possible to define the adjacency matrix 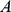 of 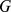 as
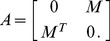
(24)


It is also useful to define the co-occurrence matrices 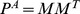 
and 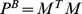 that respectively count the number of common neighbors between two vertices of 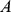 or of 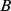. 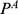 is the weighted adjacency matrix of the co-occurrence graph 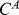 with vertices on 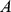 and where each non-zero element of 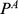 corresponds to an edge among vertices 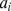 and 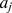 with weight 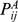. The same is valid for the co-occurrence matrix 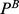 and the co-occurrence graph 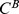.

Many projection schemes for a bipartite graph 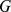 start from constructing the graphs 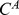 or 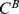 and eliminating the edges whose weights are less than a given threshold or whose statistical significance is low.

### Matrix from RCA

To make a fair comparison between the exports, it is useful to employ Balassa’s Revealed Comparative Advantage (RCA) [15] i.e. the ratio between the export share of product 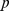 in country 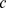 and the share of product 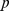 in the world market
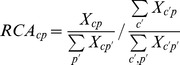
(25)where 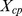 represents the dollar exports of country 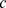 in product 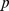.

The network structure is given by the country-product adjacency matrix 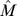 defined as
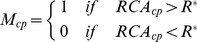
(26)where 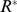 is the threshold. A positive entry, 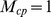 tells us that country 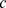 is a competitive exporter of the product 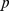.

### Minimal Spanning Forest

The spanning forest algorithm (SFA) is a computationally less-demanding variant of the Spanning Tree Algorithm (STA) where single operations can take up to 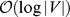 respect to the STA case where all operations are 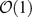. Here cluster is a synonymous for connected component.

To analyze the performance of the SFA, we use as a benchmark a weighted network with well defined communities. We consider the graph 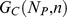 composed joining 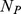 communities each consisting in a clique of 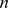 nodes; the total number of nodes is 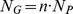. A function 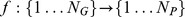 associates to each node 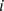 its community 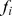; links between nodes 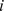 and 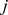 have weight 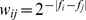. Thus, links inside a community have weight one, while links among separate communities have smaller weights. We also consider the extremely noisy case 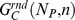 where weights between nodes 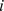 and 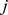 are random variables uniformily distributed in the interval 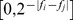.

Furthermore we shall also consider for a weighted graph 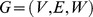 the associate threshold graph 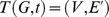 where 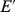 is the subset of edges in 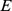 having weight higher than the threshold 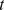. The threshold graph 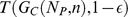 corresponds to the separated 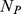 communities for 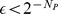.

Finally, to compare the minimum spanning forest 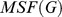 with a threshold graph 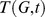, we consider the overlap 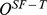 to be the fraction of links in 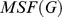 that belong to the same cluster of 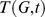.

In the non-random case, the SFA individuates correctly the communities and 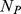 equals the number of clusters 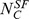 of 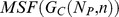. Notice that the ratio 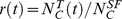 between the number of clusters 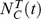 of 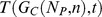 versus the threshold 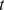 intersects the overlap 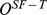 when 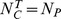 is the correct number of communities. The left panel of Fig. 6 shows such behavior for 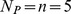.

In the noisy case, we find that 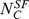 overestimates 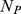; on the other hand, 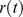 intersect 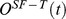 at 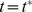 for values 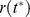 less than one and 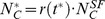 gives a better estimate of 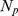. Such an effect is shown in Table 1 that shows for several values of 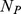, 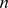 the proximity of 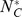 to the expected number of communities 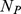. The right panel of fig. 6 shows the intersection of curves for 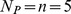 in the noisy case.
